# Determinants for the effectiveness of implementing an occupational therapy intervention in routine dementia care

**DOI:** 10.1186/1748-5908-8-131

**Published:** 2013-11-07

**Authors:** Carola ME Döpp, Maud JL Graff, Marcel GM Olde Rikkert, Maria WG Nijhuis van der Sanden, Myrra JFJ Vernooij-Dassen

**Affiliations:** 1 Radboud University Medical Center, Scientific Institute for Quality of Healthcare (IQ healthcare), Nijmegen, Netherlands; 2 Radboud University Medical Center, Radboud Alzheimer Centre, Nijmegen, Netherlands; 3 Department of Rehabilitation, Radboud University Medical Center, Nijmegen, Netherlands; 4 Department of Geriatrics, Radboud University Medical Center, Nijmegen, Netherlands; 5 Department of Primary and Community Care, Radboud University Medical Center, Nijmegen, Netherlands; 6 Kalorama Foundation, Beek-Ubbergen, Netherlands

**Keywords:** Determinants, Barrier, Facilitator, Implementation, Dementia, Occupational therapy, Psychosocial

## Abstract

**Background:**

A multifaceted implementation (MFI) strategy was used to implement an evidence-based occupational therapy program for people with dementia (COTiD program). This strategy was successful in increasing the number of referrals, but not in improving occupational therapists’ (OTs) adherence. Therefore, a process evaluation was conducted to identify factors that influenced the effectiveness of the MFI strategy.

**Methods:**

A mixed-method approach of qualitative and quantitative research was used to evaluate the implementation process. The MFI strategy as planned and as executed were reported and evaluated based on the framework of Hulscher et al. (2003; 2006). Data on OTs attitudes and expected barriers were collected at baseline from 94 OTs using a 19-item questionnaire. Data on the experiences were collected after finishing the implementation using focus groups with OTs and telephone interviews with physicians and managers. For quantitative data, frequencies and correlations were calculated and qualitative data were analyzed using inductive content analysis.

**Results:**

The implementation strategy as executed had a stronger focus than planned on increasing OTs promotional skills due to an initial lack of referrals. This resulted in less attention for increasing OTs’ skills in using the COTiD program as initially intended. At baseline, OTs had a positive attitude toward the program, however, 75% did not feel experienced enough and only 14.3% felt competent in using the program. Focus groups and interviews revealed various determinants that influenced implementation. Most managers were positive about the program. However, the degree of operational support of managers for OTs regarding the implementation was not always adequate. Managers stated that a well-defined place for occupational therapy within the dementia care network was lacking although this was perceived necessary for successful implementation. Several physicians perceived psychosocial interventions not to be in their area of expertise or not their responsibility. All professionals perceived inter-professional collaboration to be a facilitator for effective implementation, and general practitioners were perceived as key partners in this collaboration. However, collaboration was not always optimal. OTs indicated that increasing the referral rate was most effective when promoting OT via other disciplines within a physician’s network.

**Conclusion:**

Our data suggests that a first step in successful implementation should be to make sure that individual and organizational barriers are resolved. In addition, implementation should be network-based and encourage inter-professional collaboration. Initial promotion of COTiD should focus on physicians that have a positive attitude toward non-pharmacological interventions.

## Introduction

There are many innovations that have been proven effective in the research setting. However, these beneficial effects often do not reach clinical practice due to existing barriers in knowledge transfer [1,2] and implementation [3]. An example is the community occupational therapy program for people with dementia and their caregivers (COTiD). This is a client-centered program that includes 10 one-hour, home-based sessions that aims to increase or maintain independence, participation, and quality of life of people with dementia and their caregiver [4]. This intervention was found to be effective in a Dutch sample regarding clients’ daily functioning, caregiver competence and the quality of life, general health, and mood of both the client and caregiver [5-7]. Up to now, occupational therapists (OTs) have been trained in using the COTiD program through a three-day postgraduate course, including lectures, role playing, and feedback on videotaped cases [8]. Upon evaluation only 20% of the trained OTs therapists used the COTiD program or parts of it in practice [Van Uden and Graff, 2007, unpublished observations].

As a first step to increase the uptake of COTiD, barriers and facilitators to its implementation in clinical practice were evaluated [9]. Barriers found were a lack of knowledge on the COTiD program in all professionals, a lack of referrals, a lack of experience of OTs in using the program, and a perceived lack of role models and feedback for OTs [9].

Based on these barriers and facilitators [9], a one-year multifaceted implementation (MFI) strategy was developed, aimed at OTs, physicians, and managers which are essential stakeholders in the delivery of the COTiD program [9]. Training days, outreach visits, regional meetings, and access to a web-based discussion platform and reporting system were offered to OTs in addition to the postgraduate course. Physicians and managers received information about COTiD through an educational web-site, newsletters, and at least one phone call.

A cluster randomized controlled trial (CRCT) comparing the effectiveness of the MFI strategy with the three-day postgraduate course was conducted. Data were collected at baseline, six months, and 12 months [8]. Clusters were functional units offering home-based care for people with dementia and included at least two OTs, one physician, and one manager. Although the MFI strategy resulted in significantly more COTiD referrals [10], no significant difference was found between groups regarding the degree to which OTs intended to treat clients according to COTiD (OT adherence) as measured using vignettes [Döpp et al., unpublished observations]. In addition, no differences were found between groups on client and caregiver treatment outcomes [Döpp et al., unpublished observations].

### Aims and objectives

To identify factors that affected the effectiveness of the MFI strategy we conducted a process evaluation. Research questions were:

1. How was the MFI strategy executed in practice and how did it deviate from the strategy as planned?

2. What were the attitudes and barriers experienced by OTs’ at baseline regarding COTiD and its implementation?

3. How did OTs, physicians, and managers experience the MFI strategy?

## Methods

The process evaluation was conducted alongside the CRCT. Qualitative and quantitative methods were used to monitor the MFI strategy as executed. Quantitative methods were used to evaluate OTs’ baseline attitudes and expected barriers and the exposure of professionals to the implementation strategy. Qualitative methods were used to uncover experiences of OTs, managers, and physicians with the MFI strategy.

### The multifaceted implementation strategy

The model of Grol and Wensing [11], pp. 65-91 was used to develop the multifaceted implementation strategy. A multifaceted strategy was chosen because literature suggests that these strategies are more likely to result in positive effects on professional behavior [12,13] compared to the sole use of educational strategies. The strategy was based on the barriers and facilitators identified in a previous study [9]. The implementation strategy as initially developed is described in Table 1 according to the implementation process evaluation framework of Hulscher et al. [14,15].

**Table 1:** **Description of the multifaceted implementation strategy as planned based on the framework of Hulscher et al**. [14,15]

	
***1.***	***Global typing of the implementation strategy***
1.1.	*Interventions orientated towards occupational therapists*
	(a) Dissemination of educational materials using a website aimed at occupational therapists.
	(b) Educational meetings for occupational therapists (including regional network meetings).
	(c) Outreach visits for occupational therapists.
	Interventions orientated towards physicians and managers
	(a) Dissemination of educational materials using a website and newsletters aimed at physicians and managers.
	(b) Telephone calls to managers and physicians serving as reminders and providing an opportunity to ask questions about the intervention and the implementation.
1.2.	*Organizational interventions*
	Change in the patient-reporting system by offering a web-based reporting system structured according to the steps of the COTiD program.
1.3.	*Regulatory interventions*
	Accreditation for occupational therapists who are exposed to the entire implementation strategy.
***2.***	***Target group / participants***
2.1.	*Professional status*
	The intervention is developed for occupational therapists working in private practices, nursing homes, hospitals, and mental health organizations. The multifaceted intervention was developed to reach different types of physicians including general practitioners, nursing home physicians, neurologists, and geriatricians. Lastly, aimed to reach different types of manager including direct managers, such as managers of the occupational therapy department or managers of allied healthcare services and non-direct managers, such as cluster / unit managers.
2.2.	*Interaction between participants*
	Components of the implementation strategy will be aimed at the individual disciplines. However, it is assumed that each professional is part of a functional unit existing of at least two occupational therapists, one physician, and one manager. Especially the interventions toward occupational therapists are intended to encourage therapists to interact with the managers and physicians within their functional unit. In addition, we will encourage occupational therapists within the same region to interact with each other using regional network meetings.
2.3.	*Size of the target group*
	The target groups of the implementation strategy are 36 occupational therapists, 36 physicians, and 20 managers. Educational meetings will be offered in two groups (approximately 18 per group), and regional meetings will be offered in three regions (approximately 12 OTs per region). Each educational outreach visit will be offered to all occupational therapists within one functional unit at the same time (which is assumed to be two OTs per functional unit). The website is targeted at the entire group of professionals and telephone calls will be offered to the individual physicians and managers.
2.4.	*Motivation for participation*
	Accreditation points can be obtained for both participation in the study and for completing the minimum required components of the implementation strategy (minimum requirements: two educational meetings, three regional meetings, and five coaching sessions). This is done to motivate occupational therapists. Participation of all professionals is voluntarily.
***3.***	***The ‘Implementers’***
3.1.	*Professional status*
	All components of the implementation strategy aimed at occupational therapists will be executed by two ‘implementers,’ who are expert occupational therapists in executing the COTiD program as well as in teaching about the COTiD program. Both are educated in using motivational interviewing as a coaching technique.
	A third ‘implementer’ will execute the implementation strategies toward the managers and physicians. She has a background in occupational therapy and is the researcher of this study. She is also trained in using motivational interviewing.
3.2.	*Opinion leaders*
	We suspect that the ‘implementers’ providing the strategies toward the OTs will be perceived as opinion leaders and role models as they contributed to the development and testing of the COTiD program. The ‘implementer’ that will provide the strategies toward physicians and managers is not likely to be considered an opinion leader.
3.3.	*Authority*
	The researcher who developed and tested the COTiD program initiated the implementation by requesting funding for this implementation. The funding agency (Zorg Onderzoek Nederland en Medische Wetenschappen; ZONMW) is therefore also initiator of the implementation.
***4.***	***Frequency***
	*Occupational therapists*
	1) Two educational meetings (eight hours each) will be provided at the start of the intervention period with an interval of eight weeks between meetings.
	2) Outreach visits (90 minutes each): five to seven sessions depending on the individual needs. These sessions will start after the two training days with intervals between sessions depending on individual needs (approximately six to eight week intervals).
	3) Regional network meetings (2,5 hours each): four meetings in each of the three regions will be provided with intervals of approximately 12 weeks between meetings.
	*Physicians and managers*
	1) Telephone calls (duration will vary per individual): one or two telephone calls within a one year period.
	2) Newsletters: four newsletters with intervals of approximately 12 weeks.
	The website will be continuously available from the start of the intervention.
***5.***	***Information about the innovation***
5.1.	*Type of information about the innovation or guideline*
	A prerequisite for occupational therapists for starting the implementation strategy is to complete a postgraduate course on the COTiD program. During this course all OTs should have been provided with information on the entire COTiD program.
	*Information on the innovation for occupational therapists*
	1) Educational meetings:
	- Information and skills regarding the COTiD program: practicing communication skills (role-playing)
	- Information and skills regarding implementation of the program: inventorize barriers, elevator pitch, product description, promoting the program to physicians and managers (role-playing), and instructions on using the web-based reporting system and discussion forum.
	2) Outreach visits: variation is possible, but the content of the sessions needs to be a mix of improving skills to practice according to the COTiD program and skills to implement / promote the COTiD program.
	3) Regional network meetings: variation is possible, the meetings are intended to discuss cases and difficulties experienced in using the COTiD program and promoting the program.
	*Information on the innovation for physicians and managers*
	1) Telephone calls: content can vary depending on needs of physicians and managers.
	2) Newsletters: will include information on experiences with the COTiD program of various types of professionals.
	*Information on the innovation for all professionals*
	Website: will provide information on the COTiD program and publications on the effects of the program.
*5.2.*	*Presentation form and medium*
	*Occupational therapists*
	1) Educational meetings: a mixture of lectures, discussion, and role-playing.
	2) Outreach visits: variation is possible depending on the needs of the participants.
	3) Regional network meetings: lectures and discussions will be used.
	*Physicians and managers*
	Newsletters: newsletters will be sent by email to managers and physicians.
***6.***	***Information about target group management/performance***
	Occupational therapists will be provided with verbal feedback on their performance after role-playing during the educational training days. During the educational outreach visits performance and achievements will be discussed regarding both skills in executing the COTiD program and promoting the COTiD program (by addressing the number of referrals). Physicians and managers will be provided with feedback on the number of referrals that are made in the preceding period during the telephone calls.
	No information will be provided that enables participating professionals or organizations to compare their achievements with others.

Literature on implementation and knowledge translation was used to select strategies that would decrease existing barriers. This included a comprehensive overview of implementation theories and on the development and selection of strategies to establish change [16]. Because implementation is a complex process which cannot be based on a single theory [16], our MFI strategy was based on the body of thought of a group of theories.

The most important barriers identified were a lack of knowledge on the program in all professionals, a lack of referrals, a lack of experience of OTs in using the COTiD program, and a perceived lack of role models and feedback [9]. Cognitive theories state that professionals need sufficient knowledge to assist them in decision making regarding implementation [16]. Educational theories state that professionals are more likely and motivated to change their behavior when using their own problems as a starting point (*e.g.*, [17]) and literature on knowledge transfer shows that there is not one implementation strategy that fits all [2]. Based on these assumptions knowledge for OTs was incorporated into the two training days, combined with skill training. A website and newsletters were offered to physicians and managers as an easy, not-time-consuming way to gain knowledge. To address individual problems we offered telephone calls to physicians and managers. Five to seven outreach visits were offered to OTs and were led by experienced OTs (role models) who were trained in motivational interviewing. Outreach visits were chosen because this was reported to be a successful element in implementation strategies [3].

To facilitate discussion with colleagues and create sustainable support, regional meetings were organized and access to a discussion platform was provided. An electronic reporting system was developed to guide OTs through the steps of the COTiD program.

Successful implementation of the COTiD program was found to be influenced by the contact between OTs and physicians [9], we planned to encourage OTs throughout the implementation process to promote COTiD among physicians using face-to-face contact. The importance of such relationships between professionals within a network is also stressed by social network theories [18,19].

### Recruitment and sampling

Data on attitudes and barriers were collected from all 94 OTs who participated in the CRCT. Comprehensive information on the recruitment, inclusion, and exclusion criteria of these participants are reported elsewhere [8,10].

At the end of the trial, qualitative data on the experiences of professionals with the MFI strategy were collected from a purposive sample of OTs, physicians, and managers who received the MFI strategy. OTs (n = 36) [10] were requested to participate in a focus group discussion by email. For recruitment purposes the names of 36 physicians and 20 managers were ordered using random number generation. According to these lists, professionals were approached until 12 physicians and 10 managers were willing to participate. These numbers were chosen because we expected to need around ten interviews to reach saturation [20]. We checked saturation during the analysis by investigating whether new codes were still coming up and whether there was a variety of codes covering the problem of implementing COTiD.

### Evaluating deviations from the implementation strategy as planned

The strategy as initially developed is described in Table 1. The actual execution of the MFI strategy was monitored using the framework of Hulscher et al. [14,15] that requires both qualitative and quantitative data. The implementers registered quantitative data regarding the frequency with which each component of the strategy was offered and the attendance of OTs. The research team had unlimited access to the web-based system and discussion platform to collect data on the actual use of these systems by OTs. Exposure to the newsletters and website of professionals was evaluated by adding questions on the frequency of exposure to the questionnaires of the CRCT at six and 12 months follow-up. Qualitative data on the type of strategies offered, the medium used, and the type of information provided during the various components of the strategies were registered by the implementers.

### Attitudes and barriers

At baseline of the CRCT, quantitative data were collected on OTs’ attitudes and expected barriers concerning the implementation of COTiD. A web-based questionnaire including 19 statements was used. Statements were based on the previously identified barriers [9] and on statements used in a study to evidence-based practice among Dutch OTs [21,22]. OTs were asked to rate the statements on a five-point scale running from total disagreement (1) to total agreement (5). A reminder was sent two weeks after the original request.

### Experiences of health care professionals

Qualitative methods were used to collect data on the experiences of professionals with the MFI strategy. Data were collected after completion of the CRCT on 31 December 2010. Data collection was guided by topic lists (see Additional file 1). All data was audiotaped and transcribed verbatim.

### Experiences of occupational therapists

Focus groups were chosen to collect data on the experiences of the group as a whole [23] and because it provided an opportunity for participants to interact with each other in depth. Two semi-structured focus groups were held in February 2011 that were led by an experienced and independent moderator. To verify the content the moderator summarized the discussion at the end of the discussion.

### Experiences of physicians and managers

Between March and May 2011, semi-structured telephone interviews with physicians and managers were held to collect data on their experiences in a non-time-consuming way. All interviews were performed by the same independent researcher.

### Informed consent and ethical approval

This study was conducted conform the Helsinki declaration and reviewed by the ethical committee of the Nijmegen/Arnhem region, which approved the study and decided that further approval conform the Medical Research Involving Human Subjects Act (WMO) was not necessary. All participants signed a consent form prior to data collection and audiotaping. Participants participated voluntarily and were able to quit at any time.

### Data analysis

Concepts were derived from the attitude-and-barriers questionnaire using factor analysis in an exploratory manner. Internal consistency of concepts was calculated using Cronbachs’ alpha (α). Relationships between variables were assessed using Pearson’s product moment correlation coefficient (r). An alpha level of 0.05 was used for all quantitative tests.

The qualitative data were analyzed using inductive content analysis [24] using Atlas.ti version 7. Two researchers independently coded all transcripts through line-by-line analysis using open coding. Final codes were established by comparison of the codes of both researchers and by discussing its content until consensus was reached. One of the researchers grouped the final codes into categories and verified these categories with the other researcher. Subsequently, the categories were grouped into themes. The development of these themes was guided by existing literature that states that factors influencing implementation can relate to the innovation, the user, the organization, and the socio-political context [11,18,25,26].

## Results

### Characteristics of participants who received the multifaceted implementation strategy

A total of 17 clusters received the MFI strategy, including 36 OTs, 36 physicians, and 20 managers. All OTs were women with an average age of 38.5 years (sd 10.7). OTs had been qualified for an average of 13.7 years (sd 8.9). Almost half (47.2%) of the physicians were woman. General practitioners (GPs) took up 30.6% of the group and 69.4% were medical specialists. The mean age of physicians was 49.7 years (sd 7.5). Their average experience was 22 years (sd 7.1) and 53.1% had a specialization in geriatrics. Sixty percent of the managers were female. Managers had an average age of 47.8 (sd 7.06) years. More details on the study sample are reported elsewhere [10].

### Deviations from the strategy as planned

#### Interventions offered

Although not originally planned, we decided during the study to offer physicians a one-time outreach visit with the aim to increase involvement in the implementation process and visibility of the OT.

The use of the web-based system as an alternative to the OTs’ current reporting system was so problematic that coaches did not encourage its use as originally planned.

#### Target group

Although we intended to include two OTs within each functional unit, thereby making them able to support each other, two functional units included only one OT. Three functional units did not include a manager. In addition, the combination of people within 19 functional units (eight control clusters and 11 experimental clusters) varied over time [10].

Interaction between professionals within a functional unit was encouraged using all components of the MFI strategy. Actual interaction between professionals was not monitored, however, interviews with managers revealed that OTs did seek collaboration with physicians to promote COTiD. Managers mentioned that the amount of energy it took depended on the physicians (lack of) pre-existing knowledge, the type of physician, and the physicians’ target group.

#### Frequency and exposure

The number of educational and regional meetings for OTs were offered as planned (see Table 1). Educational meetings were held in two groups (group 1: 15 OTs / group 2: 21 OTs). For efficiency reasons, regional meetings were held in two instead of three regions. All OTs were offered seven outreach visits and the average number of OTs per visit was two, as planned (range, 1 – 3). The average interval between sessions was eight weeks as planned. However, great variation existed between clusters with interval periods varying from two to 30 weeks. Table 2 shows the actual exposure of OTs to the various components of the MFI strategy.

**Table 2:** Exposure of Occupational Therapists to components of the implementation strategy

	**N (%) (n = 36)**
Training days	
0 days	3 (8.3%)
1 day	2 (5.6%)
2 days	31 (86.1%)
Coaching on the job	
0 sessions	2 (5.6%)
2 sessions	1 (2.8%)
3 sessions	3 (8.3%)
4 sessions	3 (8.3%)
5 sessions	7 (19.4%)
6 sessions	9 (25%)
7 sessions	11 (30.6%)
Regional meetings	
0 meetings	2 (5.6%)
1 meeting	1 (2.8%)
2 meetings	2 (5.6%)
3 meetings	15 (41.7%)
4 meetings	16 (44.4%)
Discussion platform	
Made use of this medium:	
Yes	16 (44.4%)

We were able to reach 27 physicians (67.5%) and 18 managers (78.3%) by telephone. The average duration of telephone contacts was 15 minutes for both physicians (SD 7.0) and managers (SD 4.7). Newsletters were sent as planned. A total of 23 physicians (n = 25) and 14 managers (n = 15) read at least one newsletter. The website was visited at least once by 12 physicians (n = 20) and 13 managers (n = 16). Finally, six physicians (15%) agreed to an outreach visit that took between 30 and 60 minutes.

#### Information about the intervention

The educational meetings and outreach visits focused more on promoting OT within the OTs’ network than initially planned. This was caused by a lack of referrals in most functional units and the difficulties that OTs experienced in promoting their services. Due to this shift of focus, little time was spent on improving OTs’ skills to work with the COTiD program.

Telephone conversations with physicians mainly focused on the inclusion of people with dementia in the trial. The outreach visits for physicians were provided depending on physicians’ preference: the physician invited multiple colleagues and the researcher gave a presentation on COTiD, followed by a discussion (n = 2), or the OT and the researcher met only with the physician and presented and discussed COTiD (n = 4).

#### Attitudes and barriers of OTs at baseline

The attitudes and expected barriers of OTs at baseline of the CRCT are shown in Table 3. Three concepts were found: attitude toward COTiD (α = 0.72); experience, skills, and self-efficacy of the OT (α = 0.72); and support from the professional environment (α = 0.50).

**Table 3:** Attitudes and barriers of occupational therapists regarding the implementation of the COTiD program

**Statement**	**M (SD)**	**Totally agree N (%)**	**Agree N (%)**	**Not agree or disagree N (%)**	**Disagree N (%)**	**Totally disagree N (%)**	**V / M**
Attitude toward the COTiD program (α = 0.72)†							
It takes too much time to familiarize myself with the working method of the COTiD program. ‡	3.18 (0.77)	1 (1.8)	9 (16.1)	25 (44.6)	21 (37.5)	0 (0)	56 / 38
It takes too much time to treat clients according to the COTiD program. ‡	3.22 (0.83)	0 (0)	13 (23.6)	18 (32.7)	23 (41.8)	1 (1.8)	55 / 39
I find treatment according to the COTiD program too intensive for my clients. ‡	3.42 (0.69)	0 (0)	3 (5.5)	29 (52.7)	20 (36.4)	3 (5.5)	55 / 39
I find treatment according to the COTiD program too intensive for caregivers. ‡	3.52 (0.69)	0 (0)	2 (3.6)	27 (48.2)	23 (41.1)	4 (7.1)	56 / 38
The program provides sufficient guidance to treat people with dementia and their caregivers.	3.68 (0.77)	5 (8.9)	33 (58.9)	13 (23.2)	5 (8.9)	0 (0)	56 / 38
The intensive diagnostic phase of the program enables me to better shape the treatment.	3.85 (0.62)	6 (10.9)	36 (65.5)	12 (21.8)	1 (1.8)	0 (0)	55 / 39
Experience, skills, and self-efficacy of the occupational therapist (α = 0.72) †							
I have sufficient experience with the COTiD program.	2.07 (0.87)	1( 1.8)	2 (3.6)	11 (19.6)	28 (50)	14 (25)	56 / 38
I feel competent in using the COTiD program.	2.64 (0.82)	0 (0)	8 (14.3)	24 (42.9)	20 (35.7)	4 (7.1)	56 / 38
I find it difficult to change my old habits concerning the diagnostic phase. ‡	3.02 (0.95)	1 (1.9)	19 (35.8)	12 (22.6)	20 (37.7)	1 (1.9)	53 / 41
I find it difficult to change my old habits concerning the treatment phase. ‡	3.27 (0.84)	0 (0)	13 (23.2)	16 (28.6)	26 (46.4)	1 (1.8)	56 / 38
I feel capable of changing the procedures regarding dementia occupational therapy care at my place of work.	3.71 (0.78)	5 (8.9)	36 (64.3)	9 (16.1)	6 (10.7)	0 (0)	56 / 38
I find it difficult to justify the use of the COTiD program toward physicians. ‡	3.5 (1.03)	1 (1.8)	12 (21.4)	8 (14.3)	28 (50)	7 (12.5)	56 / 38
Knowledge of occupational therapists							
I have insufficient knowledge about dementia to be able to work with the COTiD program. ‡	3.45 (1.03)	2 (3.6)	10 (17.9)	11 (19.6)	27 (48.2)	6 (10.7)	56 / 38
Support from the professional environment (α = 0.50) †							
Role models are lacking. ‡	2.70 (1.06)	5 (8.9)	25 (44.6)	10 (17.9)	14 (25)	2 (3.6)	56 / 38
I have sufficient opportunities to ask for feedback.	3.0 (0.97)	0 (0)	17 (30.4)	13 (23.2)	17 (30.4)	3 (5.4)	56 / 38
I do not feel supported in using the COTiD program by occupational therapists at my work place. ‡	3.98 (1.04)	2 (4.2)	3 (6.3)	5 (10.4)	22 (45.8)	16 (33.3)	48 / 46
I feel supported in using the COTiD program by occupational therapy colleagues in my region.	2.8 (1.26)	3 (5.4)	18 (32.1)	12 (21.4)	11 (19.6)	12 (21.4)	56 / 38
Management at my work place supports working according to the COTiD program.	3.66 (0.72)	3 (5.4)	35 (62.5)	15 (26.8)	2 (3.6)	1 (1.8)	56 / 38
I feel supported in using the COTiD program by physicians.	2.84 (0.91)	2 (3.6)	11 (19.6)	21 (37.5)	20 (35.7)	2 (3.6)	56 / 38

**†** Internal consistency of the concept based on Cronbach’s alpha; ‡ Negatively stated items: reversed scoring system applies; V / M = valid and missing responses; M = mean; SD = standard deviation.

#### Attitude of OTs

Overall the attitude of OTs toward the COTiD program at baseline was positive. Most respondents (67.8%) agreed that the program offered sufficient guidance to treat people with dementia and their caregivers (see Table 3).

#### Knowledge, skills, and self-efficacy

Most OTs (58.9%) found that they had sufficient knowledge about dementia to work with the COTiD program. However, 75% of the respondents did not think they had sufficient experience with the program and only 14.3% felt competent in using the program. OTs who felt they had sufficient experience with COTiD felt more competent in using the program (r = 0.55; p < 0.001) and were more likely to feel capable in justifying using the program toward physicians (r = 0.37; p < 0.01).

#### Support

More than half of the OTs missed the presence of a role model (53.5%), 79.1% felt supported by their OT colleagues at work, and 67.9% felt supported by their management. Opinions varied regarding the other statements (see Table 3).

#### Relations between statements of different concepts

We found several significant correlations between items of the three concepts. OTs who agreed that they had sufficient experience with the COTiD program were less likely to experience the COTiD program to be too intensive for clients (r = 0.49; p < 0.001) and caregivers (r = 0.45; p < 0.001). OTs who did perceive the program to be too intensive for the client and caregiver also tended to experience difficulties in changing old habits in the diagnostic phase (r = 0.43; p = 0.001 and r = 0.48; p < 0.0001). In addition, OTs who felt they had sufficient experience were less likely to miss role models (r = 0.38; p < 0.01). Finally, perceived management support positively correlated to the perceived ability to justify working according to the COTiD program toward physicians (r = 0.36; p < 0.01) and OTs who missed role models were more likely to find it difficult to change old habits (r = 0.44; p = 0.001).

#### Experiences of health care professionals

In the focus groups 16 female OTs participated (three hospital (h), ten nursing home (nh), two private practice (pp), and one mental health organization (mh)). In the telephone interviews 12 physicians participated (two h, five nh, three general practice, and two mh) of which 8 were woman. Finally, 10 managers participated in the interviews (three h, five nh, and two mh) including four women.

Initially the main question was to solely collect data on the experiences of OTs, physicians, and managers with the MFI strategy. However, the interviewed professionals also provided valuable information on their experiences with the implementation of the COTiD program in general. Because this was considered useful data in explaining the effectiveness of the MFI strategy, all data were included in the analysis and presented in this paper.

Because no new codes were found and codes covered all predefined themes, data saturation was judged to be sufficient. An overview of the categories, codes, and representative quotes within each theme are provided in Additional file 2, Additional file 3 and Additional file 4. Additional file 5 includes an overview of the determinants per professional group.

### Theme one: Factors related to the implementation strategy

#### Method of dissemination

We used three methods to disseminate information: website, email with newsletter, and telephone contact. Although some physicians and managers preferred the use of email, others stated that an overflow of emails in general caused them not to read the newsletters. A benefit of email mentioned by managers was the ability to easily forward information to other professionals.

The website was perceived as useful, although both physicians and managers mentioned that it required an active attitude, which was a barrier to visit the website. Managers stated that the continuous availability of the website positively influenced its use.

Telephone calls were perceived as successful by physicians because they increased involvement and because of their ability to meet individual needs. Managers also experienced the contact moments by telephone to be positive, because it gave them an opportunity to ask questions. Planned telephone conversations were preferred.

In general, physicians preferred personal contact either by phone or during face-to-face meetings.

Almost all managers and physicians perceived the emails, newsletters, and telephone calls as reminders. Most physicians reported that the reminders made sure occupational therapy stayed on their minds when exploring a client’s options for further treatment. Managers thought the combination of different methods worked as a reminder and encouraged them to act on it.

#### Organizational factors of the implementation strategy

For some OTs it was unclear what to expect of the components of the MFI strategy, due to poor communication between the implementers and the OTs. Another experience was the extensive amount of travel time required to attend the two training days and the regional network meeting. Some suggested that future participation of OTs in regional meetings would depend on the added value of these meetings. Finally, OTs felt that the implementation period of the study should have been longer. They state that only after the study was finished the number of referrals started to increase and that they could start focusing on improving their skills.

#### Usability of the web-based system and discussion platform

OTs hardly used the web-based reporting system because it was not compatible with the way of reporting within their organizations, resulting in additional work. Even so, some OTs used the system as a training tool offering them guidance in using the COTiD program. Also, technical difficulties were experienced when working with the web-based reporting system and the discussion platform, due to flaws in the system and OTs’ poor computer skills. Finally, some OTs reported that they had doubts about the security of client information when inserted into the web-based system.

#### Focus of the implementation strategies

OTs reported that outreach visits mainly focused on improving their skills to promote the COTiD program due to an initial lack of referrals. Because of this lack OTs felt they were not able to optimally benefit from the outreach visits. OTs felt positive about the combined focus on both promotional and treatment skills and the focus of coaching on individual problems. OTs valued the educational and regional meetings most because of the opportunity to hear about experiences of OT colleagues. According to some physicians, the telephone calls were mainly focused on the inclusion of clients in the study and other matters related to the trial.

#### Added value of the implementation strategy

Managers’ opinions on the added value of the newsletters varied from clear, informative, and relevant to being of little additional value, lack of news value, and too general. They perceived the information on the website as helpful to clarify differences between OTs and other disciplines, such as case management. However, some managers missed information about the current situation regarding the nationwide implementation. One manager mentioned that not all information was relevant because his background was not in healthcare. Although managers and physicians thought telephone contact was beneficial, some managers mentioned that it did not provide new information with additional value.

#### ‘The big stick’

OTs perceived the implementation strategy to be ‘the big stick.’ Outreach visits and regional meetings ensured that OTs maintained an active attitude toward the implementation process. However, some OTs felt the implementation strategy did not contain enough obligatory aspects, which made it too easy not to work on changing their behavior. One of the suggestions made was to use the web-based system as an obligatory training tool.

### Theme two: Factors related to the COTiD program

#### Added value of OT services

It was not clear to physicians and managers how services offered by OTs differ from services offered by other professionals such as case managers, psychologists, and/or social workers. Managers especially perceived overlap with regard to interventions aimed at the caregiver. They stated that this overlap may result in competition because some organizations receive a predefined budget to spend on services with a particular aim. When deciding on this, OTs and case managers have to compete with each other. The final decision depends on the added value of occupational therapy related to other disciplines.

### Theme three: Factors related to the professional

#### OTs’ experiences with the COTiD program

OTs perceived that experience in executing the program is essential for successful implementation. Most OTs felt they needed more time to gain sufficient experience and perceived the implementation period as being too short.

#### Familiarity of physicians with occupational therapy

Physicians stated that familiarity with OT was a facilitator in increasing the chance of referrals. Several physicians that were familiar with OT prior to the study stated that this was a reason for them not to visit the educational website or perceive the telephone calls to be useful.

#### Physicians’ exposure to the COTiD population

Some physicians stated that a barrier to sending more referrals was a lack of a population eligible for treatment according to the COTiD program. The reason given most often was having a relatively young population.

#### Role of the physician within the system

Several physicians stated that they have limited knowledge and expertise regarding psychosocial interventions. One physician said that the role of physicians in dementia care is limited to Some physicians referred all people with dementia to a case management organization because they thought the case manager is more qualified to refer people to other services. One physician mentioned that making physicians aware of the effectiveness and usefulness of non-pharmacological interventions is a necessary facilitator for a successful implementation.

#### Degree of involvement and support of managers

Most managers mentioned that OTs in their organization did not require support regarding implementation. Managers relied on the input of OTs in their decision making concerning the content of the COTiD program. Managers indicated various barriers to providing sufficient support. These barriers were OTs being more experienced; the managers’ self-perceived lack of knowledge on the content of OT; managers’ perception of OTs as being independent and self-steering; managers’ faith in the knowledge and commitment of OTs; the lack of problems in the implementation; managers having many priorities; and managers having a great number of people and departments to support.

#### Managers’ attitude toward the COTiD program

Various managers were proponents of home-based OT and / or the COTiD program, because they perceived it as particularly useful for the treatment of people with dementia and their caregivers. This motivated managers to facilitate care according to this program.

#### Self-perceived role of managers

The roles of facilitator, advocate, and discussion partner were identified. Most managers perceived that their task was to facilitate OT according to the COTiD program by providing time, secretarial support, and other means necessary to make sure OTs are able to do their work. Managers mentioned that they have a role in advocating OT. This could be within the organization itself toward other managers or when negotiating with insurance companies. Finally, some managers took on the role of discussion partner during the implementation process by brainstorming with OTs on methods for implementing or promoting the COTiD program.

#### Managers’ needs

In addition to the information provided by the implementation strategies, managers felt the need for additional or other information, such as information about the development of the implementation at a national level, practical tools for the implementation process (*e.g.*, brochure on the COTiD program), and facts and figures on the implementation to help promote OT. In addition, they felt a need for information from the OTs or their organization, such as information on the progress of the implementation, on the size of the target group of the innovation, and information on the implementation in their region.

#### Factors related to professionals influencing exposure to the implementation strategy

OTs reported that commitment positively influenced their attendance to regional meetings. However, barriers to their participation in or use of the implementation strategy were a lack of familiarity with the implementation tools and having other meetings to attend to. OTs’ preference for discussing problems with their colleagues within the same organization appeared to be a barrier for using the discussion platform.

Most physicians and managers read the newsletters and website quickly. A barrier for physicians to visit the website and read newsletters was existing familiarity with OT. Facilitators for managers to visit the website were a felt need for guidance on promoting the program and feeling the need to stay informed about the activities of the OTs. If managers had questions about the COTiD program this also facilitated the use of the website. Barriers for managers to visit the website were the limited involvement in the content of OT services, no perceived need for additional information, no questions about the implementation, and having other priorities.

### Theme four: Factors related to the organization

#### Balance between cost and benefits

A major barrier mentioned by OT and managers for all aspects of the implementation strategy was the pressure of the organization to preserve a certain balance between direct time (patient care) and indirect time (*e.g.*, reporting, meetings, and education). This barrier was experienced by both OTs working within an organization and OTs owning a private practice.

#### Available capacity

The available capacity for providing home-based OT influenced the implementation of the COTiD program. One manager mentioned that due to promotion of the COTiD program they received more referrals to OT overall. This led to a fear of too many referrals than could be handled and slowed down further promotional activities. Managers reported that there are several factors that determine the available number of hours of home-based OT according to the COTiD program. These factors were the available budget; available number of trained OTs; the size of the region a department needs to cover; the demand for OT; the focus of the organization; attitude toward COTiD of other managers in the organization; effect of the intervention; and the balance between cost and benefits in clinical practice. A facilitator in several organizations was the fact that dementia was a primary area of interest of the organization and/or the region.

Several managers reported that the organizations provided OTs with additional time to participate in the study, resulting in sufficient capacity. However, this capacity tended to decrease again after completion of the study and created a lack of capacity for executing home-based OT after completion of the study.

#### Degree of collaboration between professionals

Managers perceived collaboration between professionals and departments within the organization as important. They also stated that the presence of OTs at multidisciplinary meetings in which cases were discussed was found to be helpful in increasing the number of referrals.

#### Factors related to the organization influencing exposure to the implementation strategy

OTs reported a high workload and a lack of a calm working environment as barriers preventing them from using the web-based system. Similarly, physicians did not visit the website and did not read the newsletters due to a lack of time and high workload. Some managers reported that they did not visit the website or read the newsletters because they were sufficiently informed by the OTs within their organization.

### Theme five: Factors related to the social system

#### Referral structure and local network

OTs mentioned that the lack of physicians with a sufficient amount of clients with mild to moderate dementia negatively influenced the number of referrals. Also, OTs mentioned that promoting OT using other disciplines within the physicians’ network (*e.g.*, case managers, district nurses, and physician assistants) was more effective in increasing the number of referrals than directly targeting physicians.

#### Contact between physicians and occupational therapists

In most cases, there was no frequent contact between professionals, because it was not perceived to be necessary. However, physicians who met face-to-face with OTs said this had a positive effect on their knowledge and increased awareness of the product ‘occupational therapy’.

#### Position of OTs within regional dementia care networks

Dementia care networks consist of healthcare organizations within a specific region that collaborate with the aim to synchronize care and improve the quality of care in the region. Managers said the position of the OT in these networks needs to be well assessed when creating a regional dementia network. Moreover, it needs to be clear what OTs can offer and what their areas of expertise are. One manager stated that OTs are often consulted too late and that it would be more beneficial to get involved in an earlier stage of the disease. Connections between the managers’ own organization and OTs from private practices were also seen as beneficial when the organization did not have enough OTs available to answer the demand.

#### Degree of Collaboration with general practitioners/physicians in the region

Managers perceived GPs as the most important professionals in the implementation process, especially with the Dutch healthcare system shifting from hospital-based care to more community-based care. Some managers stated that promoting occupational therapy among GPs resulted in closer working relationships and a better comprehension of each other’s work. The use of face-to-face contact and content-driven arguments in promoting OT was perceived as most useful by managers. Suggestions for further implementation were to inform other professionals within the GP’s network, such as the home health professional, nurse practitioners, case managers, and physical therapists and to use success stories to promote OT according to the program.

#### Finances and reimbursement

Reimbursement of home-based OT was not uniformly organized in the various types of organizations. Although, in general, clients can get 10 hours of home-based OT reimbursed, some organizations made separate arrangements with insurance companies. Costs of OT according to the COTiD program and especially the balance between cost and benefits were used to base final decisions on regarding the implementation. An additional barrier to implementation mentioned by managers was that the reimbursement for home-based OT was perceived to be too low to cover 100% of the cost.

#### Perceived benefits of the multifaceted implementation strategy

OTs reported an increase in awareness of the importance of promotional activities, an increase in knowledge of the COTiD program, and an increase in their motivation, energy, and execution of the program. Physicians reported an increased awareness and knowledge of the COTiD program. One physician included OT as a standard item on a checklist for further treatment options while in another organization OTs were now involved in the multidisciplinary meetings. Finally, managers stated that the telephone calls encouraged them to undertake actions such as informing healthcare professionals (including physicians) about the COTiD program and discussing the implementation process with the OTs.

#### An overview of determinants on the implementation of the COTiD program

**Figure 1:**
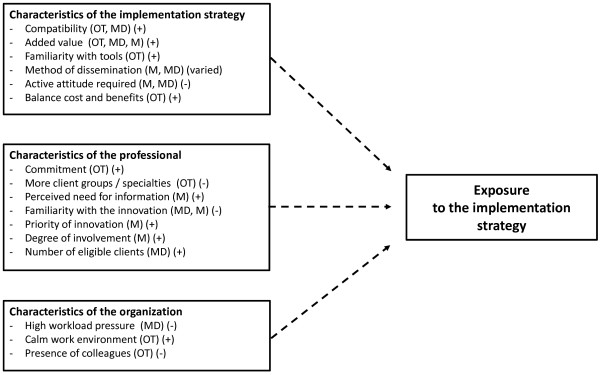
**Graphical overview of factors influencing the exposure of professionals to the implementation strategy as identified through qualitative methods.** + = positive influence on exposure; - = negative influence on exposure; Varied = direction of influence varied per individual; OT = occupational therapist; MD = physician; M = manager; qualitative data .

**Figure 2:**
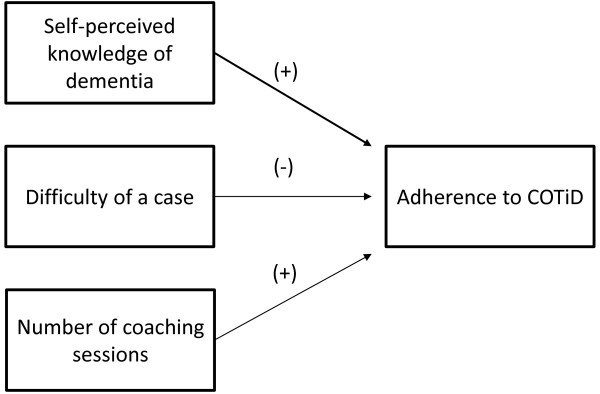
**Graphical overview of factors determining the degree of occupational therapists’ adherence to the COTiD program as identified in the effect study.***(+) = positive influence on hours OT; (-) = negative influence on hours OT; quantitative data*.

**Figure 3:**
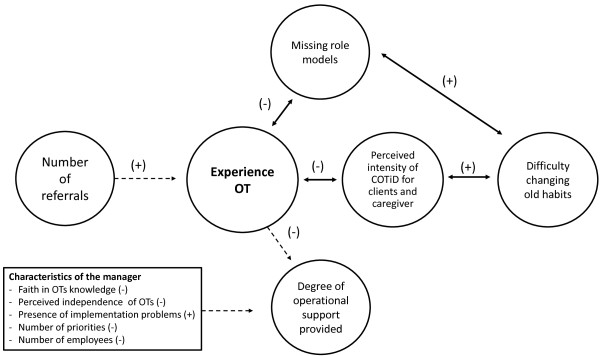
**Graphical overview of the role of experience in the implementation process and factors affecting the (self-perceived) experience.** (+) = positive relationship; (-) = inverse relationship; quantitative data ; qualitative data .

**Figure 4:**
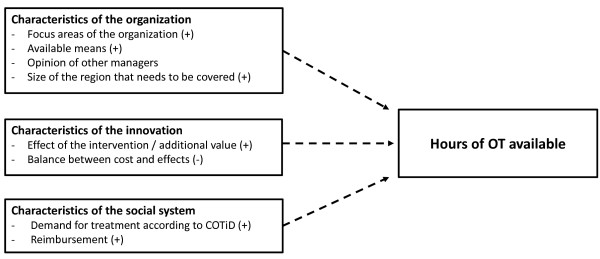
**Graphical overview of factors influencing the hours of community occupational therapy an organization has available.** (+) = positive influence on adherence; (-) = negative influence on adherence; qualitative data .

Upon evaluation of all data regarding the implementation of COTiD, various connections between determinants and between effects and determinants were found. Figures 1, 2, 3 and 4 provide a quick overview of these findings. Exposure to the implementation strategy is a prerequisite for the strategy to have any effect. Figure 1 shows that we found various determinants that affected this exposure.

Having sufficient experience with, as well as sufficient adherence to the COTiD program are assumed to be essential to establish effective implementation. Figures 2 and 3 show how various determinants affect experience and adherence. The hours of occupational therapy made available may also influence the degree of implementation. Figure 4 summarizes our finding that factors regarding the innovation, the organization, and the social system may affect the available hours of OT.

## Discussion

Implementation of effective interventions into the healthcare system is complex. Our process evaluation showed that the main focus on increasing OTs promotion and network skills may both explain the significant increase in the number of referrals in the intervention group [10] and the lack of effect on OT adherence and client and caregiver treatment outcomes. However, both OT adherence and the referral rate were still low. Additional determinants identified in this process evaluation provide guidance to improve the MFI strategy. These determinants also provide useful guidance for the development and implementation of other complex interventions within healthcare. This study contributes to strengthening the body of knowledge on implementation in the area of allied healthcare [27] where only a limited number of implementation studies have been conducted [27-34].

### Essential components of implementation strategies

#### Organizational prerequisites

Before an individual professional can adopt an innovation the organization needs to adopt it first [18]. Organizations should evaluate the feasibility of implementation for the organizations itself and for the individual professionals. The focus areas of the organization, the available means, and the demand for the program should be considered on organizational level. The number of referrals, the number of specialties, and the requirements regarding patient-related time should be taken into account regarding the OTs feasibility. Also, a competent leader who provides sufficient leadership support is essential in creating an effective team [22,35] and is associated with a higher quality of care [36]. However, most managers we interviewed provided their OTs with a lot of freedom and limited support. To facilitate implementation in the long run, the development of an organizational structure to facilitate and monitor implementation of evidence-based interventions is recommended [37].

#### Collaboration and contact between professionals

Collaboration between professionals was found to be an important element which is in line with a review [38] that found that interdisciplinary collaboration may positively affect professional behavior. This process evaluation showed that especially interpersonal contact helped to increase physicians’ knowledge and awareness of OT. This is in agreement with previous literature suggesting that interpersonal contact is important, especially with people who are less open to change [18]. In our MFI strategy, we only indirectly encouraged OTs to collaborate with physicians in their region and the exposure of physicians and managers to interpersonal communication seemed limited. Studies regarding multifaceted implementation strategies suggest that a multidisciplinary and network-based approach is both feasible [32] and effective [39] in improving attitudes, knowledge, and behavior [40]. This indicates that a more prominent place for interdisciplinary collaboration within our strategy might have been necessary to kick-start collaboration and networking. Future implementation studies should also look into the role of interpersonal contact and include the frequency and content of interpersonal contact as an outcome measure.

#### Position within a regional network and selection of partners

Collaboration within a regional network was found to be important. Managers and physicians stated that it was not always clear to them how occupational therapy differed from services offered by other professionals. This indicates that it is important that professionals clearly communicate about their services and its additional value to professionals within their network.

In addition, our results suggest that careful selection of physicians to collaborate with may enhance implementation. Based on our findings we recommend that OTs focus their promotional efforts on physicians who fall under the categories ‘early adopters’ and ‘early majority’ because they can be used to increase the speed of implementation [18]. These physicians should who have a sufficient number of clients and have a positive attitude toward psychosocial interventions. This approach may limit broad-based implementation, but can be seen as an initial step to establish short-term implementation. To establish a more broad-based implementation in the long run a next step could be to identify and use opinion leaders from the group of professionals from the first step. However, even though this seems a promising method of implementation, more studies should be conducted to the use and effects of local opinion leaders as concluded by Flodgren et al. [41].

Managers perceived GPs to be the most important professionals to focus implementation efforts on. This is in agreement with the opinion of the Dutch College of GPs, which assigns the GP a primary role in dementia care management [42]. To ensure implementation of effective interventions this role requires additional support GPs. An example of effective support strategies are the use of pre-approved referrals [43] or the use of automated referrals [44].

#### Strengths and Limitations

We identified various factors that contributed to the success and failure of the implementation of a complex intervention that are useful for research and practice. A limitation is that the initial goal of the interviews was to solely reflect on the experiences with the MFI strategy. However, other relevant factors to the implementation process were identified and reported. Because the interviews were not prepared based on this broader goal the overview of determinants may not be exhaustive. Finally, most OTs were of the opinion that the implementation period (one year) was too short to establish change in the number of referrals as well as their skills in using the program. Future studies should carefully select the intervention and study period. A balance should be found between providing sufficient time and maintaining momentum [11].

## Conclusion

Implementation of effective innovations is complex. Our MFI strategy was effective in increasing the number of referrals to the COTiD program. However, it was not effective in increasing OT adherence. The feasibility of implementation for both the organization and individual professionals should be evaluated first. To facilitate the implementation process a competent managers and an organizational structure that facilitates implementation and monitoring are necessary. When continuing the implementation process, collaboration between professionals within a regional network is important and preferably takes place through interpersonal contact. Physicians to collaborate with should be selected based on sufficient number of eligible clients and their attitude toward the intervention.

## Competing interests

The authors state they have no competing interests.

## Authors’ contributions

CD was the investigator and designed the study, supervised data collection, analyzed the data, and wrote the manuscript. MG and MVD acquired funding for the study. MG, MVD, RN, ST, and MOR were responsible for the design, project supervision, and writing. MG also contributed to the data analysis of the qualitative data. All authors critically read and approved the final manuscript.

## Supplementary Material

Additional file 1 **Topic guides for focus groups and telephone interviews.** Provides information on the topic lists used for the focus groups with occupational therapists and telephone interviews with physicians and managers. Click here for file

Additional file 2 **Themes, categories, and codes from the focus groups with occupational therapists.** This file provides a table in which all themes, categories, and codes are displayed that were extracted from the focus groups data. In addition, for each category several representative quotes are given. Click here for file

Additional file 3 **Themes, categories, and codes from the interviews with physicians.** This file provides a table in which all themes, categories, and codes are displayed that were extracted from the data collected using the interviews with physicians. In addition, for each category several representative quotes are given. Click here for file

Additional file 4 **Themes, categories, and codes from the interviews with managers.** This file provides a table in which all themes, categories, and codes are displayed that were extracted from the data collected using the interviews with managers. In addition, for each category several representative quotes are given. Click here for file

Additional file 5 **Determinants per theme of implementing the COTiD program as identified by each professional group via focus groups and interviews.** Provides a summary of all factors found that may have affected the implementation of the COTiD program, which are presented for each professional group and per theme. Click here for file
