# Supplementary material for: Determinants for the effectiveness of implementing an occupational therapy intervention in routine dementia care
Source: Implement Sci. 2013 Nov 7;8:131. doi: 10.1186/1748-5908-8-131 (PMC4226207; doi:10.1186/1748-5908-8-131)
Supplement: Additional file 1 — Topic guides for focus groups and telephone interviews. Provides information on the topic lists used for the focus groups with occupational therapists and telephone interviews with physicians and managers. [file 1748-5908-8-131-S1.pdf]

Additional file 1 – topic guides for focus groups and telephone interviews

1. Topic guide for focus groups with occupational therapists

- Experiences with the educational meetings
  (E.g.: duration, usefulness, ability to meet needs, organization and coordination, execution of the meetings, additional value for clinical practice)

- Experiences with the outreach visits
  (E.g.: duration, usefulness and effect on daily practice, number of sessions, ability to meet needs, organization and coordination, execution, relation with coaches)

- Experiences with the web-based reporting system
  (E.g. used or not?, compatibility with current method of reporting, user-friendliness, additional value)

- Experiences with the regional meetings
  (E.g. content of the meetings, meet professional needs, usefulness of the information and discussions, opportunity for networking, frequency, organization, execution, duration)

- Experiences with the discussion platform
  (E.g. user-friendliness, usefulness, additional value, reasons for using or not using the discussion platform)

- Additional value of the multifaceted implementation strategy compared to the postgraduate course.
  (E.g. what was the additional value? What strategies contributed most to personal development of the professional? Was it worth the time investment?)

- Suggestions for improving the multifaceted implementation strategy.
  (E.g. what would you change regarding the implementation strategy? What should stay the same?)

2. Topic guide for telephone interviews with physicians

- **General information**
  - *How is the contact between you and the occupational therapists that participated in the study?*
  - *Do you have patients that are eligible for treatment according to the COTiD program? [people with dementia and their caregiver who are living at home]*

- **Experiences with the educational website**
  - Opening question: *Did you visit the website on the COTiD program?*
  - If not, ask the physician what the reason was for not visiting the website.
  - If the website was visited: What did you think about the website? (I.e. usefulness of the information, degree to which needs are met, relevance of the different subjects discussed, lay-out, user-friendliness)
  - *What effect did the information from the educational website have on your knowledge about occupational therapy for people with dementia and your professional behavior? (I.e. the number of people you refer or the type of people you refer?)*

- **Experiences with the newsletters**
  - Opening question: *Did you read one or more newsletters?*
  - If not, ask what the reason was for not reading the newsletter.
  - If newsletters were read: What did you think about of the newsletters*?* (I.e. degree to which needs are met, influence on (referral) behavior, number of newsletters, method of disseminating newsletters, lay-out)
  - *What effect did the information from the newsletters have on your knowledge about occupational therapy for people with dementia and your professional behavior? (E.g. the number of people you refer or the type of people you refer?)*

- **Experiences with the telephone contacts?**
  - Opening question: *Did the researcher contact you by telephone at least once?*
  - If so, *what did you think about these contact moments?* (I.e. satisfaction with answers to questions, number of contact moments, effect on (referral) behavior, effect on knowledge of occupational therapy for people with dementia)
  - *What effect did the telephone contacts have on your knowledge about occupational therapy for people with dementia and your professional behavior? (E.g. the number of people you refer or the type of people you refer?)*

- **Additional value of the multifaceted implementation strategy for daily practice.**
  - *What strategy / components of the implementation strategy had the least effect on you knowledge and daily practice? And why?*
  - *What strategy / components of the implementation strategy had the most effect on you knowledge and daily practice? And why?*

- **Suggestions for improving the multifaceted implementation strategy**
  - *What suggestions do you have for improving the website, newsletters and telephone contact?*
  - *What should be done to (further) increase your knowledge or to change you referral behavior?*

3. Topic guide for telephone interviews with managers

- **General information**
  - *What is your relationship with the occupational therapists that participated in the study?* [operational manager or cluster/unit manager]
  - *To what extent do you have influence on making OT according to the COTiD program available in your organization?* [In other word: are you in a position to facilitate OT according to the COTiD program?]

- **Experiences with the educational website**
  - Opening question: *Did you visit the website on the COTiD program?*
  - If not, ask the manager what the reason was for not visiting the website.
  - If the website was visited: *What did you think about the website?*
    (I.e. usefulness of the information, degree to which needs are met, relevance of the different subjects discussed, lay-out, user-friendliness)
  - *What effect did the information from the educational website have on your knowledge about occupational therapy for people with dementia and your professional behavior?* (I.e. the way you supported the OTs in their work?)

- **Experiences with the newsletters**
  - Opening question: *Did you read one or more newsletters?*
  - If not, ask what the reason was for not reading the newsletter.
  - If newsletters were read: What did you think about of the newsletters*?*
    (I.e. degree to which needs are met, influence on behavior, number of newsletters, method of disseminating newsletters, lay-out)
  - *What effect did the information from the newsletters have on your knowledge about occupational therapy for people with dementia and your professional behavior?* (I.e. the way you supported the OTs in their work?)

- **Experiences with the telephone contacts?**
  - Opening question: *Did the researcher contact you by telephone at least once?*
  - If so, *what did you think about these contact moments?*
    (I.e. satisfaction with answers to questions, number of contact moments, effect on behavior, effect on knowledge of occupational therapy for people with dementia)
  - *What effect did the telephone contacts have on your knowledge about occupational therapy for people with dementia and your professional behavior?* (I.e. the way you supported the OTs in their work?)

- **Additional value of the multifaceted implementation strategy for daily practice.**
  - *What strategy / components of the implementation strategy had the least effect on you knowledge and daily practice? And why?*
  - *What strategy / components of the implementation strategy had the most effect on you knowledge and daily practice? And why?*

- **Suggestions for improving the multifaceted implementation strategy**
  - *What suggestions do you have for improving the website, newsletters and telephone contact?*
  - *What should be done to (further) increase the implementation of occupational therapy at home for people with dementia and their caregivers and what is your role in this implementation process?*
