# Supplementary material for: Determinants for the effectiveness of implementing an occupational therapy intervention in routine dementia care
Source: Implement Sci. 2013 Nov 7;8:131. doi: 10.1186/1748-5908-8-131 (PMC4226207; doi:10.1186/1748-5908-8-131)
Supplement: Additional file 2 — Themes, categories, and codes from the focus groups with occupational therapists. This file provides a table in which all themes, categories, and codes are displayed that were extracted from the focus groups data. In addition, for each category several representative quotes are given. [file 1748-5908-8-131-S2.pdf]

Additional file 2 - Themes, categories, and codes from the focus groups with occupational therapists

Theme	Category	Codes	Representative Quotes
Factors related to the implementation strategy	Focus of the implementation strategy	Focus on referrals; focus on barriers regarding skills; focus on individual barriers; good mix of content and promotion; when more referrals focus shifts to content; little to discuss due to lack of referrals; RM_recognition and affirmation; RM_exchange experiences; EM_recognition of experiences other OTs; EM_affirmation of practice	"…a lot to do with how to get these referrals, it had a lot to do with that. How to get in touch, how to get people to come to you, what do you have to do to refer the right patients. And concerning treatment, indeed some confirmation but personally I didn't get down to the treatment a lot. "<br><br>"For me a very positive thing was the combination of a focus on content and on PR, referrals, logistics, that kind of thing and that is the kind of combination you need, to get something started, it was a very good thing that there was a focus on both these things."<br><br>"Of course it was also a question of made-to-measure and it was very much focused on how you do that kind of thing, and that was fantastic and it also helped me a great deal."
	"The big stick"	Active attitude; not enough obligations; refresher	"The coaching on the job was very good because it really gives you a sparring moment, and I always had some questions to ask but it was also always a bit noncommittal. That is also partly my own doing, but if we were told that it was an assignment you have to do that for the next meeting, well then I think you will prepare yourself better. You do need the big stick."<br><br>"Oh well, it was a nice start, really. For us the post graduate training was some time ago so for me it was a good way to brush up my knowledge so that it would become more vivid again."
	Usability of the web-based system and discussion platform	Not compatible with existing system; privacy client; technical issues; not broadly applicable; efficiency in sharing information; workload pressure	"…I notice that I don't apply it [refers to the web-based system] in practice because then you have to use it alongside the usual electronic patient file and the usual reporting, it does not serve as a replacement so you would need to spend extra time on it and there is no time for that really."<br><br>"I mostly used it in the beginning [refers to the web-based system] for the structure of the treatment, what steps to take, what information"<br><br>"I did study it a few times at the beginning but to be honest, I'm really not into computers. We do have a laptop for primary care, so that we can report straight away when we are at the client's home and I did manage to make myself familiar with that but apart from that, the internet and that kind of thing…"

	Organizational factors of the implementation strategy	Communication on the goal of the implementation strategies; travel time; collaboration within the region; duration of the implementation period	"I was actually hoping to learn new things but that didn't happen [refers to the educational training days]. I went over there all enthusiastic, thinking this is great, learning new things but it wasn't new at all. I thought that was a real pity. Perhaps if the word 'consolidate' was used at an earlier stage it would have put us on the right track a little sooner"<br><br>"It should really take longer, you know. There are two parts in the research, and the first part was easy to comply with, the multiplication of clients and PR but then, proving that your ability had improved, well yes, that was complicated."<br><br>"Which made me think at some point, alright, that means that I'll have to travel for two hours for a regional meeting lasting two hours and then I'll have to travel back for two hours. For me that did stand a bit in the way rather."
Factors related to the occupational therapist	Experience with the innovation	Experience prerequisite for implementation; insecure due to lack of experience	"You want to implement that guideline, partly because of the coaching, am I doing it right and how, how do I market it. But it is also routine, practicing it a lot."
	Factors related to professionals influencing exposure to the implementation strategy	Commitment of OT; OT_lack of familiarity with tools; OT_treating a variety of client populations	"In my opinion, you decided to join this so then you go for it. So for me the production was not at first a reason to say that I wouldn't be joining them."<br><br>"I would really have to think very seriously about what things I am going to make an effort for, because we've got this primary care group, we've got the ParkinsonNet, which is six times a year. Which makes me think at some point: when do I get to see my patients?"
Factors related to the organization	Balance between cost and benefits	Requirements production influence time for implementation activities; participation in courses increases work load pressure; implementation course takes up a lot of indirect hours; monitoring the balance between direct and nondirect time; course selection selective due to production requirements	"We put up quite extensive reports, which takes up a lot of reporting time. And when you are doing an AMPS (assessment of motor and process skills), that also takes up a lot of indirect time. At one point you get a sort of imbalance, how much direct and indirect time did we spend on it. It's a continuous search."<br><br>"You do have to consider that because you have to achieve your production, you have to consider which meeting you will attend and which you won't attend."
	Factors related to the organization influencing exposure to the implementation strategy	High workload pressure; lack of calm working environment	"We put up quite extensive reports, also for the geriatrician, which takes up a lot of reporting time. And when you are doing an AMPS, that also takes up a lot of indirect time. At one point you get a sort of imbalance, how much direct and indirect time did we spend on it. It's a continuous search."

Factors related to the socio-political context	Referral structure and local network	Promotion via other disciplines; referral via physician is a barrier; implementation via primary care; unsuitable physician; difficulties in referral from an organization to primary care services; variation in referral structure between settings; PR difficult due to changes of assistant physicians	"Well, in my own hospital it was also an issue that it was thought that eventually it [refers to the COTiD program] should not take place in the hospital, this care, but from primary care from a nursing home."<br><br>"the really useful people are counselors and district nurses who visit people at home, perhaps the GPs"<br><br>"….every three months there was a different assistant physician at the memory outpatient clinic, so that was no help either."

OT = occupational therapist; RM = regional meetings; EM = educational meetings
