# Supplementary material for: Determinants for the effectiveness of implementing an occupational therapy intervention in routine dementia care
Source: Implement Sci. 2013 Nov 7;8:131. doi: 10.1186/1748-5908-8-131 (PMC4226207; doi:10.1186/1748-5908-8-131)
Supplement: Additional file 3 — Themes, categories, and codes from the interviews with physicians. This file provides a table in which all themes, categories, and codes are displayed that were extracted from the data collected using the interviews with physicians. In addition, for each category several representative quotes are given. [file 1748-5908-8-131-S3.pdf]

Additional file 3 - Themes, categories, and codes from the interviews with physicians

Theme	Category	Codes	Representative Quotes
Factors related to the implementation strategy	Method of dissemination	Electronic most effective; preference for email; email efficient; preference for mail; read quickly due to full email inbox; too much email in general; preference for information on paper; method of dissemination influences exposure; newsletter suitable for general information; website requires active attitude; prefers verbal information; phone calls more forceful; phone calls increase involvement; phone calls and email complementary; phone calls most effect on knowledge and skills; preference for face-to-face contact; phone calls better fits need for information; outreach visit most effective; phone calls least effective.	"….it is more a case of receiving so much email that I really have to be critical in what to read and what not, because my inbox is always filled to overflowing, which is why it is not high on my list of priorities" (physician, female, mental health organization)<br><br>"….the website really is the place where all the information can be found. Anything you want to know, you can find it there. But it is something that you have to do yourself, actively. And the other things [refers to newsletter and telephone contact] will come to you automatically" (physician, female, nursing home)<br><br>"….I was pleased that she [the researcher] was able to inform our team about it [the COTiD program]. Last year I indicated that I took part in the study in my team. But well, at that time you don't know all the ins and outs yet." (physician, female, mental health organization)<br><br>"It [the contact by telephone] is probably a more direct link to your need for information."(general practitioner, male)
	Focus of the strategies	Telephone contact mainly focused on recruitment of clients; website: good overview of OT services	"I think it [telephone contact] was more a discussion on how to include clients" (general practitioner, female)
Factors related to the innovation	Added value of OT services	Perceived overlap OT and psychologist and social worker; perceived overlap between OT and social work; OT most suitable for making a snapshot of patient functioning; OT more relevant with the shift of services to the home-environment	"Well alright, again I do perceive a certain kind of overlap between the role of case management where it concerns influencing the system or advising, supporting, counseling or instructing the informal caregiver, which leads me to think who does what exactly." (physician, male, hospital)<br><br>"The role of case management is to counsel, to explain the clinical picture and to give advice in dealing, and to support people with emotional behavioral problems. That does indeed cause overlap with OTs, who also aspire to this. So we don't approach ET for that kind of thing." (physician, male, nursing home)
Factors related to the physician	Role of the physician within the system	OT outside medical domain; OT not in medical domain and therefore not in physicians' mind; prerequisite	"So it isn't at the forefront of your mind at all and then there is a last complicating factor which is the nature of occupational therapy, I mean it's allied to medicine and not medical. It's not an immediate concern in the

		implementation: awareness of usefulness non-pharmacological interventions; role limited to referring; non-pharmacological interventions do not belong to the physicians' stock; case managers more suitable for referring people	perception of the physician, which makes it also a more intangible thing." (physician, male, hospital)<br><br>"…a prerequisite for that would have to be to try and make people like me and in my position more aware of the relative value of such a thing [OT according to COTiD]. Doctors are after all simply doctors. What they like to do most is give medication or apply plasters and talk to the patient. All those kinds of behavioral therapy or modalities, they are not really part of a physician's stock, and before a doctor is ready to start attending to or using this very broad arsenal, well I think that will take quite some time." (physician, male, hospital)<br><br>"Doctors tend to think in doctor's terms, doctor's illnesses, doctor things and doctor's performances, and interventions that are aimed at a type of psycho education, well those are more outside the medical domain. Of course you can feel that it is perhaps a good thing and a useful thing. And if that is really the case and all, but well, as I said it's rather outside our cognitive domain." (physician, male, hospital)<br><br>"And I also think that it is better done through case management or those docteams which we are using now, I mean nursing home physicians or geriatricians who primarily visit and counsel people at their homes. I think they will be more receptive to the possibilities of such an approach." (physician, male, hospital)
	Familiarity of physicians with occupational therapy	Read literature before start of the study; used to working with OT; little contact with OT prior to study	"Well yes, it's in, it's part of my framework so to speak, in my frame of mind. It's just that, whenever it is needed an OT always comes to mind because I am used to working with OTs." (physician, female, mental health organization)
	Physicians' exposure to the COTiD population	Patient population not suitable for COTiD; not enough eligible patients	"….my practice consists of rather younger patients." (general practitioner, female)<br><br>"What I have been doing now is mainly trying to refer patients from the geriatric day care clinic. What I noticed especially was that a number of people were declining cognitively and did not fit into the treatment anymore, or that relatives indicated that there were so many healthcare professionals working there that they don't want any more people involved. So in practice it turned out to be quite difficult to really find people to refer." (physician, female, nursing home)

	Factors of the professionals influencing exposure to the implementation strategy	Setting priorities; website not visited; only visited website at the start; rough view of website; read newsletter; did not read newsletter; roughly read the newsletters; took a quick look at the newsletters; did not use website_familiair with OT	"I just think at the time I need it then I think of OT because I'm used to working with OTs. So I don't really need to visit the website to find out if it would be suitable or not. I've just never found a good reason to visit the website." (V99)
Factors related to the organization	Factors of the organization influencing exposure to the implementation strategy	Workload pressure; did not visit website due to workload pressure; did not read newsletters due to work load pressure; workload pressure suppresses active attitude; no need for phone calls due to work load pressure; website no priority; setting priorities; newsletter no priority	"I really don't have the time for that, I really can't fit that into my working schedule." (answer to the question whether she visited the website) (physician, female, mental health organization)<br><br>"With all this workload pressure you're not likely to actively visit a website." (physician, female, nursing home)<br><br>"I think it's to do with pressure of work as well. I think it will be good when there will be time and space for it in your daily structure." (physician, female, mental health organization)
Factors related to the socio-political context	Contact between physicians and occupational therapists	Good collaboration; contact OT most effective for implementation; contact through mail; contact moments limited; contact with OT positive effect on knowledge; used to working with OTs; frequent contact not necessary; visit of OTs positive effect on referrals; enthusiasm of OTs inspiring	"We had a visit from two OTs. They introduced themselves and told us what it is they do. At least that gave us a chance to get to know them by name and by face, and also to get familiar with the product. As a result, we are much more aware of occupational therapy when we are making treatment plans." (physician, male, nursing home)<br><br>"Discussing things with the occupational therapist [in answer to the question: what turned out to be most effective]. This is what we are doing at the moment and this is what we have on offer and yes, reading about it. I think that whenever a colleague is talking very enthusiastically to you about something, then it will encourage you to broaden your knowledge about it." (physician, female, nursing home)<br><br>"We are in touch with each other but we don't talk to each other on a regular basis, I think we meet up once a year, although we do run into each other in for example a nursing home. Whenever I fax something they get to work on it and they inform me about it in a pleasant way. It is really not necessary to have frequent contact. It's all running smoothly really." (general practitioner, female)

OT = occupational therapist; COTiD = Community Occupational Therapy in Dementia
