# Supplementary material for: Determinants for the effectiveness of implementing an occupational therapy intervention in routine dementia care
Source: Implement Sci. 2013 Nov 7;8:131. doi: 10.1186/1748-5908-8-131 (PMC4226207; doi:10.1186/1748-5908-8-131)
Supplement: Additional file 4 — Themes, categories, and codes from the interviews with managers. This file provides a table in which all themes, categories, and codes are displayed that were extracted from the data collected using the interviews with managers. In addition, for each category several representative quotes are given. [file 1748-5908-8-131-S4.pdf]

Additional file 4 - Themes, categories, and codes from the interviews with managers

Theme	Category	Codes	Representative Quotes
Factors related to the implementation strategy	Added value of the strategy	NL_superficial; NL_boring; NL_informative; NL_clear; NL_information no additional value; NL_little additional value; NL_most relevant for informing manager; website makes NL needless; NL_nice and compact way to provide information; information difficult for layman; website: clarified difference between OT and other professions.	"I didn't think the newsletter had a very high additional value for me." (Manager, male, mental health organization)<br><br>"I think the newsletter rather competes with the website. If the information on the website is appropriate and it is a project that has been running for a number of years, then you can ask yourself what is the value of the information in the newsletter. A good thing about the newsletter though is that it focuses attention on matters." (Manager, male, mental health organization)<br><br>"By being able to find the guideline on the website, and being able to get information it's easier for me to determine the position between those two [refers to OT and case management], we noticed some conflicting interests." (Manager, male, mental health organization)
	Method of dissemination	Too much email in general; preference for electronic dissemination; preference for email; preference for mail; website continuously available; website good as a reference; website suitable for information that does not change often; telephone calls are invasive; telephone contact creates space for managers to contact the interventionist; website requires active attitude; telephone calls makes managers less focused; interventions work as reminder	"…it does give you partly a learning moment, it is a good thing to reread things every now and then, to let it sink in more. Just reading it or hearing it once, well, that is likely to subside in the multitude of things you are dealing with. So being momentarily alerted to it does lead to remembering it." (Manager, female, hospital)<br><br>"For me that doesn't work as well as getting it via the post, because I receive more than fifty mails every day." (Manager, female, hospital)<br><br>"Yes, that was an excellent way of dissemination. In fact, all information sent digitally is better processed than when it is sent by post." (manager, male, nursing home)
Factors related to the innovation	Added value of OT services	Competition between OT and case managers; overlap between OTs and case managers; difference between OT and case manager minimal; OT additional value compared to case management; resistance of case managers	"actually ambulatory occupational therapy is a bit at odds with what we are developing now regionally for people with dementia, which is case management. You see, the aids are not done through case management but a number of things such as unburdening everyday practice for informal caregivers and also, making sure that they know about everything, that does give some overlap."<br>(Manager, male, mental health organization)

			"Well, we have to make a choice at that point, what type of professional will we use for home counseling for the patient with dementia or and/or the informal caregiver, and yes, an occupational therapist could very well be the right person for that. Although it could just as well be a case manager, so the additional value in relation to each other can only be indicated when you really do need more adaptations than just the counseling" (Manager, male, mental health organization)
Factors related to the manager	Degree of involvement and support of managers	OTs do not need support; limited guidance_experienced OTs; OTs self-steering; OTs independent; OTs did not communicate any problems; manager trusts / relies on OT regarding implementation; manager has many employees	"these people have been working as professionals for more than 25 years, there's no need to add anything to that. Of course, when an OT has only just started working, we'll have to give more intensive guidance obviously, but that was not the case here at all." (Manager, male, hospital)<br><br>"…you see if the OT had indicated that he or she had difficulties with certain things, whether it be an aspect of time or other things to do with content, well then I would have to know about it so I can act upon it but that hasn't occurred yet. These are all very experienced OTs, so no, I didn't play a role in that at all." (Manager, male, hospital)<br><br>"I get my information through the OTs and that's what we discuss. So I don't for instance study the articles, I gladly leave that to the OT, they say this is what you can or should do from the management. They can really asses it on content and they can see whether it really fits into the choices that we make here as occupational therapy group, because you can't do everything, you can't excel in everything you know, especially where developments are concerned so, what choices do we make, what will we do and what not and also the usual care, well we always go along with that of course." (Manager, male, hospital)<br><br>"If I were an operational manager, well then I would obviously communicate more about that. I would have really immersed myself into the subject but because I am a more indirect manager I am in a relationship based on mutual trust with the team. I left a lot to the occupational therapy teams and adopted a more supportive role, which was why I was not able to answer very detailed questions, questions dealing with content." (Manager, male, nursing home)<br><br>"but because we have quite a large group of allied healthcare professionals, I don't always see the people very often. So I do my

			managing from a distance.” (Manager, male, nursing home)
	Self-perceived role of managers	Role of facilitator; allow home-based treatment; manager needs to be aware of what OTs are working on; support_promoting OT; support_advocate for OT; support_provide OTs with time; support_money; determine target areas for policy; sparring partner	“…the content is on the employees’ desk. And to support them when needed, where it concerns means and materials so that they can indeed do their job properly, not only within their project but also within care. You see, information that is important for me is, but that has to come from the hospital itself, the size of the target group and what type of effort is required here, and then it’s my turn to move and do I have those means at my disposal or not and if not how, how do I get them, how do I try to acquire those means. That is especially my part you know, staff and means.” (Manager, male, hospital)<br><br>“It’s all about whether the managers who are involved actually bring up the subject or whether the insurer or the care administration office brings up the subject. That’s a subject I could bring up.” (Manager, male, mental health organization)<br><br>“No, that is my most important role, to support them as much as possible in that and to brainstorm together on how to deal with this and whom to approach, yes we do that together.” (manager, male, nursing home)
	Managers’ needs	Website_lacked updates on national implementation; Website_ PR toolkit was lacking; need for information on success implementation within cluster; need for regional information; need for knowledge exchange with other clusters	“for instance when making the brochure, we’re all actually re-inventing the wheel which is really a shame, it leads to delays…..a complete PR toolkit would have been handy or at the very least a brochure, but also for instance a standard text that you could use for a newspaper article, which we ended up writing ourselves, which could be put in the newspaper, those kind of things make me realize well, that would be good if you could just take that from the newspaper, such a press report, well you can send that in such a way.” (Manager, female, nursing home)<br><br>“Well you see, if you really want to know then the information you want to get is how things go when OTs do home visits, how are OTs treated and another thing is just, do you succeed in convincing referring professionals and other professionals of the importance” (Manager, male, nursing home)<br><br>“…so I need regional information, I need information on how things are going in our area, well that always needs to originate from personal contact with the OTs themselves, for it to be successful you especially want to have experience from your own region.” (Manager, male, nursing

			home)
	Managers' attitude toward the COTiD program	Need for home-based interventions; positive about COTiD; proponent home-based interventions; enthusiasm manager important for implementation	"Well yes, it obviously has added value and you do see every time that it really helps clients and informal caregivers, that's really great of course." (Manager, female, nursing home)
	Factors related to professionals influencing exposure to the implementation strategy	NL_read globally; Website_visited when questions arose; visited website several times; website not visited; visited website at the start of the study; visits to website limited_little involvement in implementation; visited website _curious; webiste_not visited_no questions; webiste_not visited_well-informed by OTs	"…in any case when there was a reason for it, for instance when we were asked questions or when we wanted to look something up." (Manager, female, hospital)<br><br>"…whenever you have questions, you want to know how something works, what is it I can do here, well then of course you want to visit a website to get more information. I never had those questions." (M30)
Factors related to the organization	Available capacity home-based OT	Fear of too many referrals; OT limited capacity for home-based treatment; finances determine number of hours for home-based treatment; lack of trained OT to cover field of activity; realistic capacity determines choice between OT or other professional; dementia point of attention of organization; implementation COTiD in annual planning; maintaining functioning at home point of attention in the region; awareness of management of importance OT essential for implementation	"We have a dementia center for diagnosis and case management here, where OTs gave a presentation, which always leads to more referrals in other fields as well. So well, that's why you cannot give too much attention to it because it just leads to too many referrals." (Manager, female, hospital)<br><br>"Well, to find out its added value and whether its availability is realistic. I mean, it's not exactly realistic when for instance results show that occupational therapy is really the best option in ambulatory care for people with dementia, but we only have one occupational therapist available for that kind of care." (Manager, male, mental health organization)"<br><br>"The occupational therapists and I can assess the interests and the use of it ourselves, and subsequently management will have to see whether we can and should adjust the hours of occupational therapy in our formation. And in that my influence is limited. In fact, money is the decisive factor here." (Manager, female, mental health organization)<br><br>"The issue of dementia is something that is paid attention to not only within the whole organization, but also within our region. Also, reinforcing the possibility of continuing to live at home is an important factor." (Manager, male, nursing home)
	Degree of collaboration between	Collaboration with other OTs in	"…a doctor who chairs our multidisciplinary meetings has also received

	professionals	primary care; collaboration between organization and private practice possibility	information and is aware of the study. We also discussed with our therapist, our occupational therapist, the idea behind it and what she would like to do and so on, and for some time our occupational therapist also took part in our meetings to keep the subject to the fore. But when she stopped taking part in it the interest quickly died down, so I can influence it but often only at some distance” (Manager, male, mental health organization)
	Factors related to the organization influencing exposure to the implementation strategy	Website not visited_sufficient information provided by OT	“But it’s really because of the independence of my therapists and their fantastic know-how that I received all the information I needed, without having to look for further information.” (manager, male, nursing home)
Factors related to the socio-political context	Position of OTs within dementia care networks	OTs get involved too late; presence of OT in multidisciplinary meetings increases referrals; determine position of OT in dementia care network	“What we notice is that we get involved in it too late, and we feel that we should have been involved much earlier on, now patients are at the verge of being admitted and that makes you think that that could have been prevented if we had been involved right from the beginning.” (Manager, female, nursing home)<br><br>“In the coming years a care network will be developed, resulting in an agreement between now and the end of 2012. Well, that is the moment to concentrate on the position of occupational therapy in that care network.”(Manager, male, mental health organization)<br><br>“So gradually we are establishing our presence here and we are succeeding in covering the region, but the question remains who in this target group is specialized in what. And I notice that some occupational therapists are quite good at indicating their speciality, for instance fall prevention”(Manager, female, hospital)
	Degree of collaboration with physicians in the region	GP_important group of physicians; PR GPs important due to shift to primary care; Increase implementation_inform physicians via other disciplines; increase implementation_increase knowledge of people within GPs’ network; increase implementation_discuss succesful cases; suggestion implementation_focus on informing	“You see, general practitioners are of course an important, a very important group.” (Manager, male, nursing home)<br><br>“General practitioners are the focal points. Especially with the current government aiming to transfer care from the hospital to people’s neighborhoods, and if you cannot make it very clear what you have on offer, well then a GP is really not going to take you into consideration.”(Manager, female, hospital)<br><br>“Which means that the approach from home care is very important, meaning incorporating health professionals in primary care, home care

		physicians	with case managers, that I think is an ideal breeding ground for occupational therapy in dementia care. I also think that physiotherapists should focus a lot more on occupational therapy." (Manager, male, nursing home)<br><br>"Also the communication between GPs and OTs has really improved because of that *[refers to PR campaigns aimed at GPs]*, shorter lines yes, also there is more mutual understanding so that they know where to find each other, yes I'm quite convinced of that." (manager, male, nursing home)"<br><br>"If you bother GPs by saying for instance we are occupational therapists and we'll be taking this up, what often happens is that the GPs have an aversion toward it. But we used substantive arguments to bring it to their attention and that appealed to a lot of GPs."(manager, male, nursing home)<br><br>"And it is always the case in primary care that a successful referral leads to more referrals, but if you never had such a successful referral, well then you are leaving out a large area."(Manager, male, nursing home)
	Finances and reimbursement of the COTiD program	Production agreement with insurance_default reimbursement not applicable; organization determines which means are used to achieve aims; finances do not limit home-based treatment; possibility of home-based treatment determined by cost/benefits; after study no capacity for home-based treatment; balance between patient and non-patient time; rate home-based OT too low; available capacity home-based OT depends on demand	"…so we will be evaluating whether our occupational therapist could be assigned more hours to work on that, so we will have to find out to what extent the demand in the past period and in the coming period will increase. So the demand by clients, the number of applications."(Manager, female, mental health organization)<br><br>"another aspect is the rate for home-based OT, I think that is really substandard, it's an investment in time and I think that insurers should fully reimburse it." (Manager, male, hospital)

NL = newsletter; OT = occupational therapist; COTiD = Community Occupational Therapy in Dementia; GP = General Practitioner
