# Supplementary material for: Determinants for the effectiveness of implementing an occupational therapy intervention in routine dementia care
Source: Implement Sci. 2013 Nov 7;8:131. doi: 10.1186/1748-5908-8-131 (PMC4226207; doi:10.1186/1748-5908-8-131)
Supplement: Additional file 5 — Determinants per theme of implementing the COTiD program as identified by each professional group via focus groups and interviews. Provides a summary of all factors found that may have affected the implementation of the COTiD program, which are presented for each professional group and per theme. [file 1748-5908-8-131-S5.pdf]

Additional file 5 - Determinants per theme of implementing the COTiD program as identified by each professional group via focus groups and interviews

OCCUPATIONAL THERAPISTS		MANAGERS		PHYSICIANS	
Determinant of the implementation strategy					
Poor communication on the aim of the strategies	-	Method of dissemination used (e.g. email, telephone)	Varied	Method of dissemination used (e.g. email, telephone)	Varied
Extensive amount of travel time required	-	Reminder function of the strategy	+	Reminder function of the strategy	+
Balance between travel time and added value of meetings	Varied	Added value of the newsletters, website, and telephone calls	Varied	Added value of telephone calls	Varied
Too short (supported) implementation period / lack of time to get experienced with the program	-			Main focus of telephone calls on inclusion of clients in the trial	-
Lack of compatibility with clinical practice and clinicians preferences.	-				
Doubts about the security of the web-based system	-				
Main focus on improving promotional skills	+ / -				
Focus on both promotional skills and practical skills	+				
Focus of coaching on individual problems	+				
Opportunity to exchange experiences with colleagues	+				

Too little obligatory aspects	-				
Degree to which the intervention serves as a "big stick"	+				
Determinants related to the innovation					
		Perceived overlap with OT and services of other professionals	-	Perceived overlap with OT and services of other professionals	-
		Additional value of OT / effect of the intervention in clinical practice	Varied	Additional value of OT / effect of the intervention in clinical practice	Varied
		Balance between cost and effects	Varied		
Determinants related to the professional					
Lack of experience in using the COTiD program	-	Perception that OTs do not need support in the implementation process	-	Familiarity with the OT / COTiD program	+
Commitment	+	Lack of support in the implementation process	Varied	Lack of eligible clients	-
		Positive attitude toward home-based OT	+	Limited knowledge of psychosocial interventions / lack of awareness of beneficial effects	-
		Self-perceived role in the implementation process	Varied		
		Need for additional information and tools	-		
		Having other priorities	-		
Determinants related to the organization					
Organizational pressure to maintain	-	Organizational pressure for OTs to maintain	-	Work-load pressure	-

certain balance between direct (patient) time and non-direct time.		certain balance between direct (patient) time and non-direct time.			
Work-load pressure	-	Available capacity of OTs	Varied		
		Promoting the COTiD program	+		
		Available number of trained OTs	Varied		
		Size of the region the organization needs to cover	Varied		
		Available budget	Varied		
		Demand for OT	Varied		
		Focus of the organization on dementia care	+		
		Attitude toward COTiD of other managers in the organization	Varied		
		Presence of OTs in multidisciplinary meetings	+		
		Collaboration between professionals and departments	+		
Determinants of the social-political context					
Lack of physicians with sufficient number of eligible clients	-	Clear position for OTs in regional networks	+	Face-to-face contact with OTs	+
Promoting OT through other professionals in the physicians network	+	Face-to-face contact between OTs and general practitioners	+		
		Promoting OT through other professionals in the physicians network	+		

		Use of successful cases in promoting OT toward physicians	+		
		Method of reimbursement	varied		

+ / - = positive or negative influence on implementation; Varied = the direction of the effect varied depending on the respondents; OT = occupational therapist; COTiD = community occupational therapy in dementia
